# pH-Responsive Mixed Polymeric Micelles as Gel-Related Nanocarriers for Drug Delivery: A DPD Study on Block Ratio Modulation

**DOI:** 10.3390/gels12090823

**Published:** 2026-09-08

**Authors:** Wensheng Wu, Zhiwei Li, Xiang Li, Wenyuan Zeng, Zhimao Lin, Shasha Liu

**Affiliations:** School of Environmental and Chemical Engineering, Zhaoqing University, Zhaoqing 526061, China; lizhiwei@zqu.edu.cn (Z.L.); lix@zqu.edu.cn (X.L.); wenyuanzeng1@163.com (W.Z.); linmoore@163.com (Z.L.)

**Keywords:** dissipative particle dynamics, pH-responsiveness, polymeric mixed micelles, block ratio, drug release

## Abstract

Polymeric micelles represent a fundamental self-assembled architecture of gel-based soft materials and have emerged as promising nanocarriers for anticancer drug delivery. Their performance is largely governed by the block composition of constituent copolymers, and understanding their self-assembly behavior provides critical insights into the rational design of gel-related drug delivery systems. In this work, dissipative particle dynamics (DPD) simulations were performed to systematically investigate two types of mixed drug-loaded micellar systems self-assembled from a triblock copolymer mPEG-b-poly(2-(diethylamino)ethyl methacrylate)-b-PMMA (PDEAEMA, hereafter referred to as the DMA block for brevity) with either a diblock copolymer PDEAEMA-b-PMMA (polymer B) or PPEGMA-b-PDEAEMA (polymer C). By tailoring the ratios of hydrophobic (MMA, the constituent block of PMMA) and pH-sensitive (DMA) blocks, the protonation-responsive behavior, structural stability, drug loading capacity, and release kinetics of the micelles were comprehensively examined. The simulation results demonstrate that: (1) increasing the hydrophobic block ratio accelerates the protonation-triggered micellar swelling and drug release because the increased hydrophobic content enhances the core compactness which, upon protonation, generates a stronger driving force for chain extension, yet an optimal ratio (+16 MMA units) exists beyond which excessive hydrophobic blocks suppress release due to core densification; (2) increasing the pH-sensitive block ratio significantly enhances the maximum drug loading capacity (from 9.83% to 12.22% for the A/C system), but exerts only limited influence on the release rate; (3) the A/C mixed micelles with higher PEG content exhibit superior structural stability and drug loading capacity, while the A/B system with higher MMA content displays more sensitive pH-responsiveness. These findings reveal a competing mechanism between “protonation-driven force” and “structural resistance,” providing mesoscopic theoretical guidance for the rational design of pH-responsive polymeric nanocarriers and self-assembled soft materials via block ratio modulation.

## 1. Introduction

Cancer has become a major public health issue severely threatening human health, and chemotherapy remains one of the most commonly employed treatment modalities. However, anticancer drugs generally suffer from poor water solubility, low selectivity, and severe toxic side effects, which greatly limit their clinical application efficacy [1]. Polymeric micelles, as a novel class of nanoscale drug delivery vehicles, can effectively solubilize hydrophobic drugs through their unique core–shell architecture and achieve passive targeting to tumor tissues via the enhanced permeability and retention (EPR) effect [2]. Among them, pH-responsive polymeric micelles have emerged as a frontier direction in smart drug delivery systems owing to their ability to sense the pH difference between the tumor microenvironment (pH 5.0–6.5) and normal physiological environment (pH 7.4) and trigger on-demand drug release [3,4].

In recent years, the mixed micelle strategy—namely, the co-assembly of two or more different block copolymers into mixed micelles—has attracted extensive attention due to its capacity to integrate the functional advantages of individual components [5]. Mixed micelles enable the simultaneous optimization of multiple performance metrics, including high drug loading capacity, enhanced structural stability, and environmentally triggered release, by tuning the polymer composition ratios [6]. For instance, Luo et al. [7] developed pH/redox dual-responsive mixed micelles self-assembled from mPEG-b-PAE and PAE-ss-mPEG, achieving efficient encapsulation of doxorubicin (DOX) and triggered release within tumor cells. Wei et al. [8] systematically investigated the effects of different hydrophilic/hydrophobic block ratios of amphiphilic block copolymers on the self-assembly and drug release behavior of pH- and ultrasound-dual-responsive micelles, identifying the block ratio as a critical parameter governing micellar structure and performance.

In the design of pH-responsive polymeric micelles, poly(2-(diethylamino)ethyl methacrylate) (PDEAEMA, hereafter referred to as the DMA block for brevity) has been widely employed as a pH-sensitive block due to its pKa of approximately 7.0–7.4 depending on the local microenvironment [9]. For brevity and consistency with the DPD model notation, the PDEAEMA block is denoted as ‘DMA’ throughout this manuscript. This abbreviation is model-specific and should not be confused with the chemical nomenclature for dimethylamine or N,N-dimethylacrylamide. The protonated form is denoted as ‘DMAH’. When the environmental pH falls below the pKa, the tertiary amine groups of PDEAEMA undergo protonation, converting from a hydrophobic to a hydrophilic state, which triggers micellar disassembly and drug release [10]. Nevertheless, a systematic mesoscopic understanding of how the proportion of the PDEAEMA block influences the overall performance of mixed micelles—including structural stability, drug loading capacity, and release kinetics—remains lacking. Furthermore, the effects of the hydrophobic block (e.g., poly(methyl methacrylate), PMMA) ratio on the post-protonation swelling behavior and drug release rate also warrant in-depth investigation.

Dissipative particle dynamics (DPD), as a mesoscopic coarse-grained simulation method, enables substantial extension of both temporal and spatial scales of simulations while preserving intermolecular interaction information, and has thus become a powerful tool for investigating the self-assembly and drug release behavior of polymeric micelles [11,12]. In recent years, DPD has been extensively applied to studies on the morphological evolution and drug loading/release mechanisms of pH-responsive polymeric micelles [13,14,15,16]. Ma et al. [17] employed DPD simulations to investigate the pH-responsive self-assembly and drug release behavior of μ-ABC miktoarm star polymers, revealing that the lengths of both hydrophilic and pH-responsive arms exert decisive influences on micellar morphology and release performance. Feng et al. [18] assessed the structural stability of poly(ethylene glycol)-poly(L-lactic acid) (PEG-PLLA) nanoparticles with different block sequences using DPD, identifying the hydrophilic/hydrophobic block length ratio as a key determinant of nanoparticle stability. Zhou et al. [19] studied the co-assembly of amphiphilic triblock copolymers with nanodrugs and the associated drug release kinetics via DPD simulations, elucidating the regulatory mechanism of hydrophobic block length on release rate. Experimental characterization of real-time morphological changes and core densification during protonation remains challenging with conventional techniques (e.g., TEM, DLS), motivating the use of DPD simulations to probe these dynamic processes at the mesoscale with molecular-level resolution.

Despite the aforementioned advances, a systematic mesoscopic understanding of how the ratios of different functional blocks (hydrophilic, hydrophobic, and pH-responsive) in mixed micelles synergistically influence protonation-responsive behavior, structural stability, drug loading capacity, and release kinetics is still lacking. To address this gap, the present study focuses on two mixed micellar systems formed by the self-assembly of a PDEAEMA-containing triblock copolymer mPEG-b-PDEAEMA-b-PMMA (polymer A) with either a diblock copolymer PDEAEMA-b-PMMA (polymer B) or PPEGMA-b-PDEAEMA (polymer C) [20]. The A/B and A/C combinations were specifically selected to systematically vary the hydrophilic/hydrophobic balance while maintaining a constant PDEAEMA content. This design allows us to isolate and compare the effects of PEG corona thickness (in the A/C system) and MMA core composition (in the A/B system) on micellar performance, which cannot be achieved with a simple diblock micelle system where multiple variables would change simultaneously. Using DPD simulations, we systematically investigated: (1) the effects of varying the hydrophobic block (MMA) ratio on the morphological evolution and drug release of micelles before and after protonation; and (2) the effects of varying the pH-sensitive block (DMA) ratio on micellar structural stability, maximum drug loading capacity, and drug release performance. Through these investigations, we aim to elucidate the molecular mechanisms underlying block ratio modulation of the drug delivery performance of mixed micelles, thereby providing theoretical guidance for the rational design of pH-responsive mixed micelles. These findings may also inform the design of related soft material systems, such as supramolecular hydrogels, where similar block copolymers are used as building blocks.

## 2. Results and Discussion

### 2.1. Protonation-Induced Micellar Structural Transition: Effect of Hydrophobic Block Ratio

The interaction parameter a_ij_ reflects the degree of repulsion between different bead types; a smaller a_ij_ value indicates stronger attraction (or weaker repulsion) between the two components, while a larger value indicates stronger repulsion and thus a greater tendency for segregation.

To investigate the effect of hydrophobic block ratio on the protonation-responsive behavior, stable drug-loaded micellar initial structures were first obtained under neutral conditions (pH = 7.4). Subsequently, the interaction parameters between DMA and other components were replaced with those of DMAH to simulate the protonation process as the pH decreased to 5.0. Upon protonation, the interaction parameter between DMAH and water decreased significantly from 115.27 to approximately 31.19, while the interaction parameters between DMAH and other components such as MMA, PEG, and DOX increased substantially (e.g., DMAH–MMA from 25.65 to 232.19, DMAH–PEG from 36.56 to 137.94), as listed in Table 1. This variation converted the pH-sensitive block from a hydrophobic to a strongly hydrophilic state, driving its extension from the micellar interior toward the corona. This protonation-induced hydrophilic–hydrophobic transition mechanism is consistent with the pH-responsive release behavior observed in the experimental study of PDEAEMA-based mixed micelles by Chen et al. [21].

**Figure 1:**
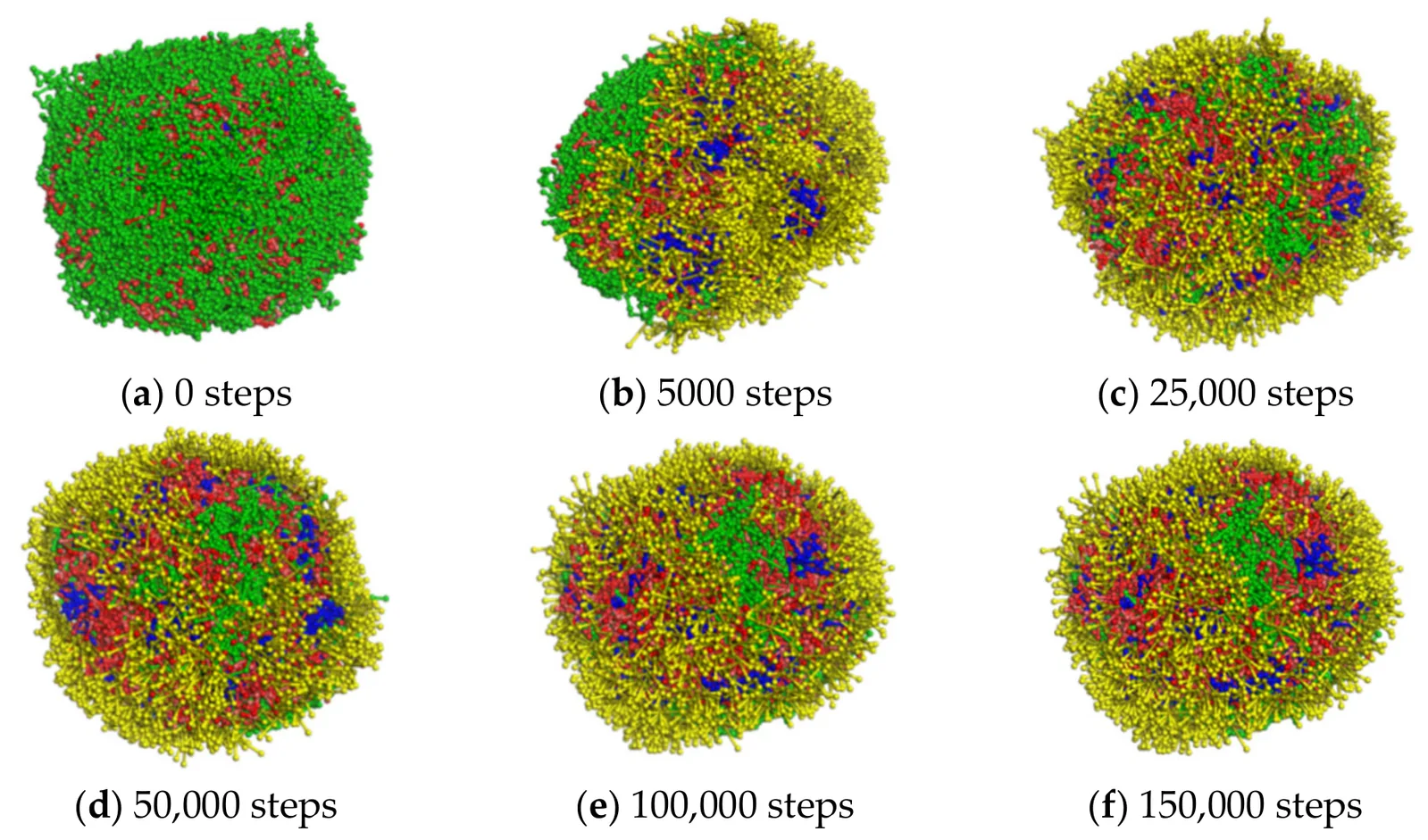
Morphological evolution of A/B mixed micelles during the protonation process.

**Figure 2:**
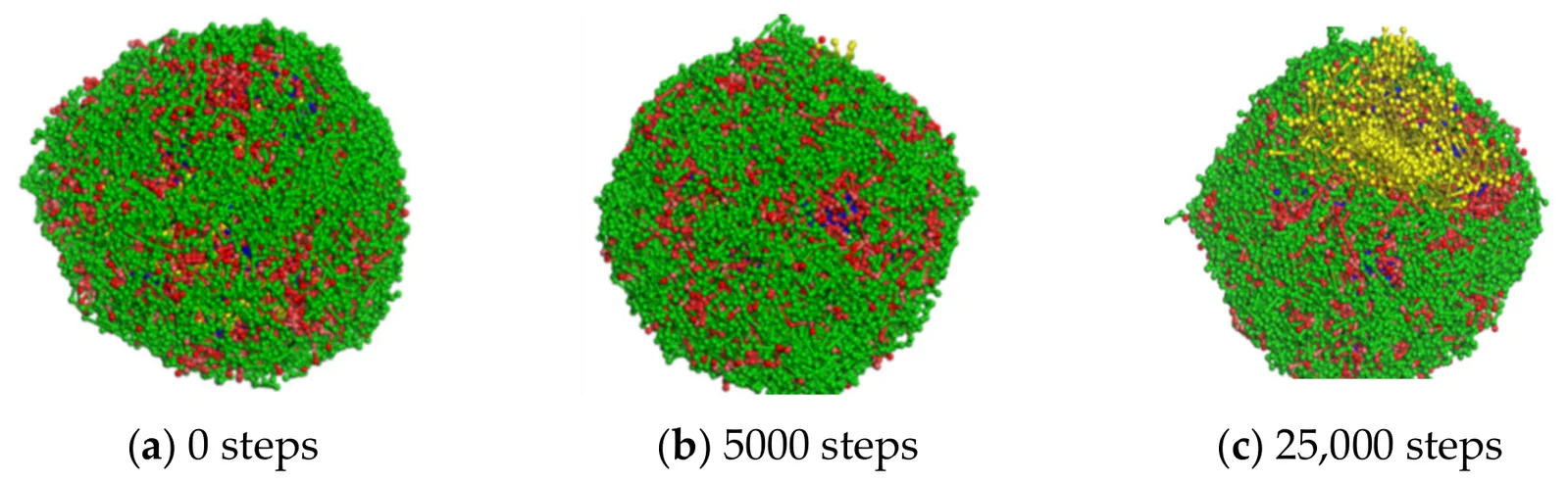

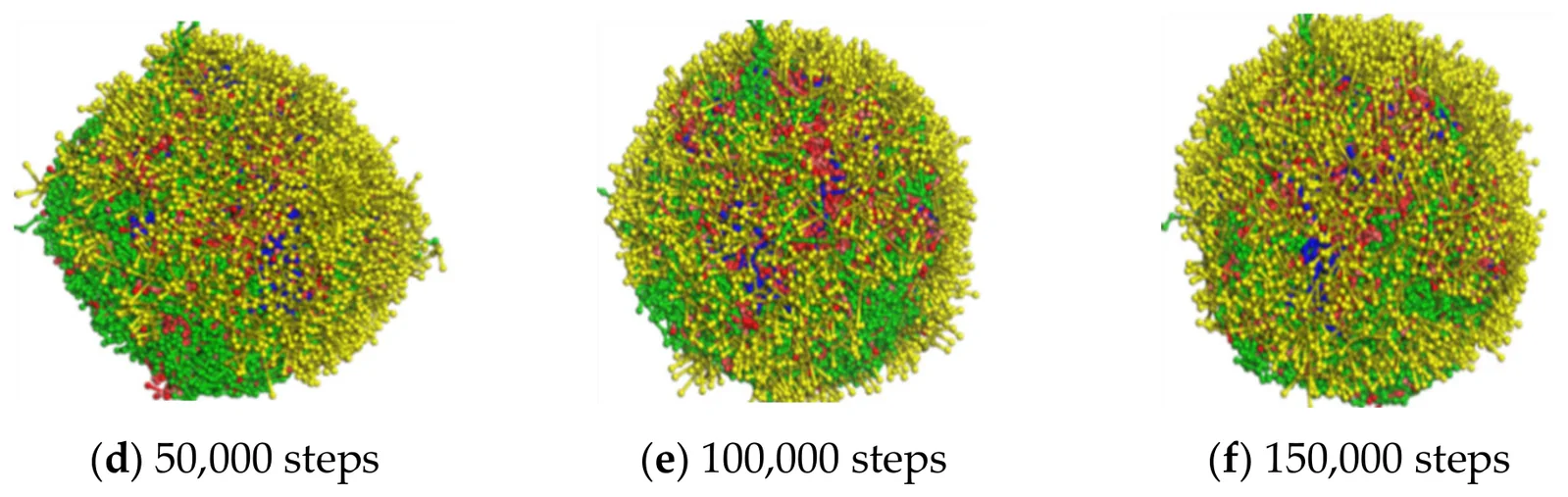
Morphological evolution of A/C mixed micelles during the protonation process.

The morphological evolution during the protonation process is shown in Figure 1 and Figure 2. At the early stage of the simulation (0–5000 steps), numerous DMAH blocks had already extended out of the micellar corona in the A/B mixed micelles, whereas only a few DMAH blocks were observed to extend out of the corona in the A/C mixed micelles. This difference indicates that the A/B mixed micelles, which possess a higher proportion of hydrophobic blocks, respond more rapidly to pH changes. The underlying reason is that the A/B system contains a higher ratio of hydrophobic MMA, resulting in a more compact micellar core, which exerts a stronger driving force for the outward extension of the protonated DMAH blocks. In contrast, the A/C system has a thicker hydrophilic PEG corona, which provides a certain buffering and hindering effect on the extension of DMAH blocks [20]. After 100,000 steps, both types of micelles reached equilibrium, with the DMAH blocks almost completely extended outside the micellar corona.

The analysis of the radius of gyration further quantitatively confirmed this trend (see Table 2): the Rg of the pH-sensitive blocks in the A/B mixed micelles increased from 19.72 before protonation to 28.25 after protonation (an increase of 8.53), while that in the A/C mixed micelles increased from 18.30 to 23.30 (an increase of 5.00). The greater extension degree of the pH-sensitive blocks in the A/B micelles is consistent with their faster response rate.

**Figure 3:**
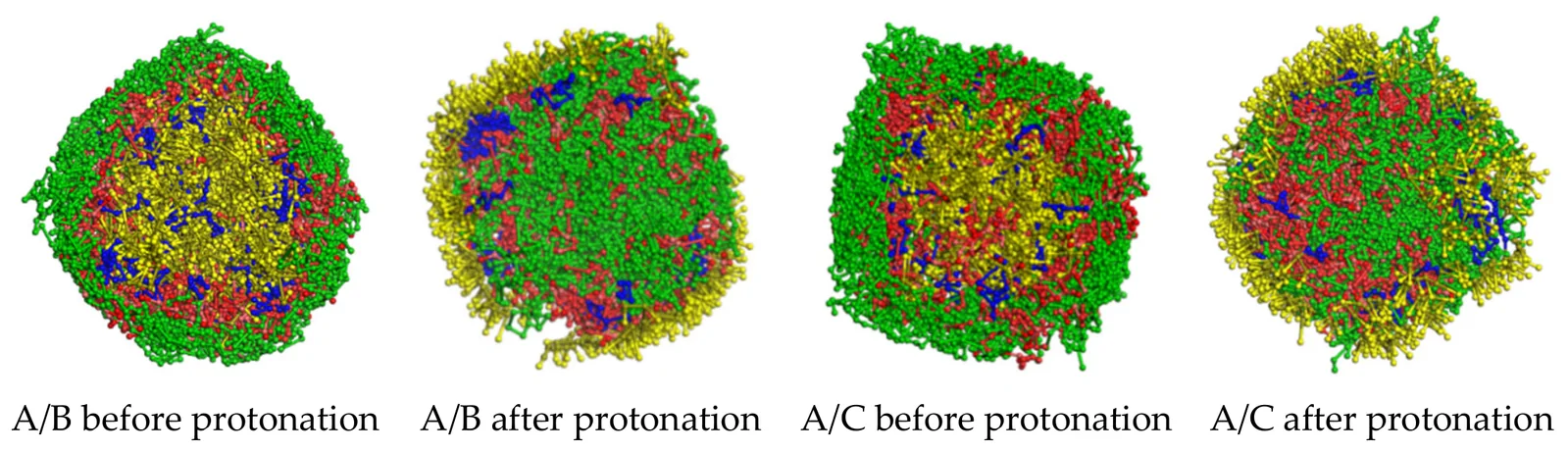
Cross-sectional views of A/B and A/C mixed micelles before and after protonation.

The comparison of cross-sectional views before and after protonation is presented in Figure 3. Before protonation, the micelles exhibited well-defined layered structures with compact organization among different components. After protonation, cracks appeared inside the micelles, the boundaries between components became blurred, and the distribution of DOX expanded significantly, almost throughout the entire micellar region. This indicates that the protonation-induced micellar swelling disrupted the original core–shell ordered structure, creating favorable conditions for drug release.

### 2.2. Effect of Hydrophobic Block Ratio on Micellar Structure and Drug Distribution

The radial distribution function g_ij_(r) measures the local density of j-beads around i-beads as a function of distance r. A higher peak intensity indicates greater accumulation of the two bead types at that distance, reflecting stronger spatial association.

**Figure 4:**
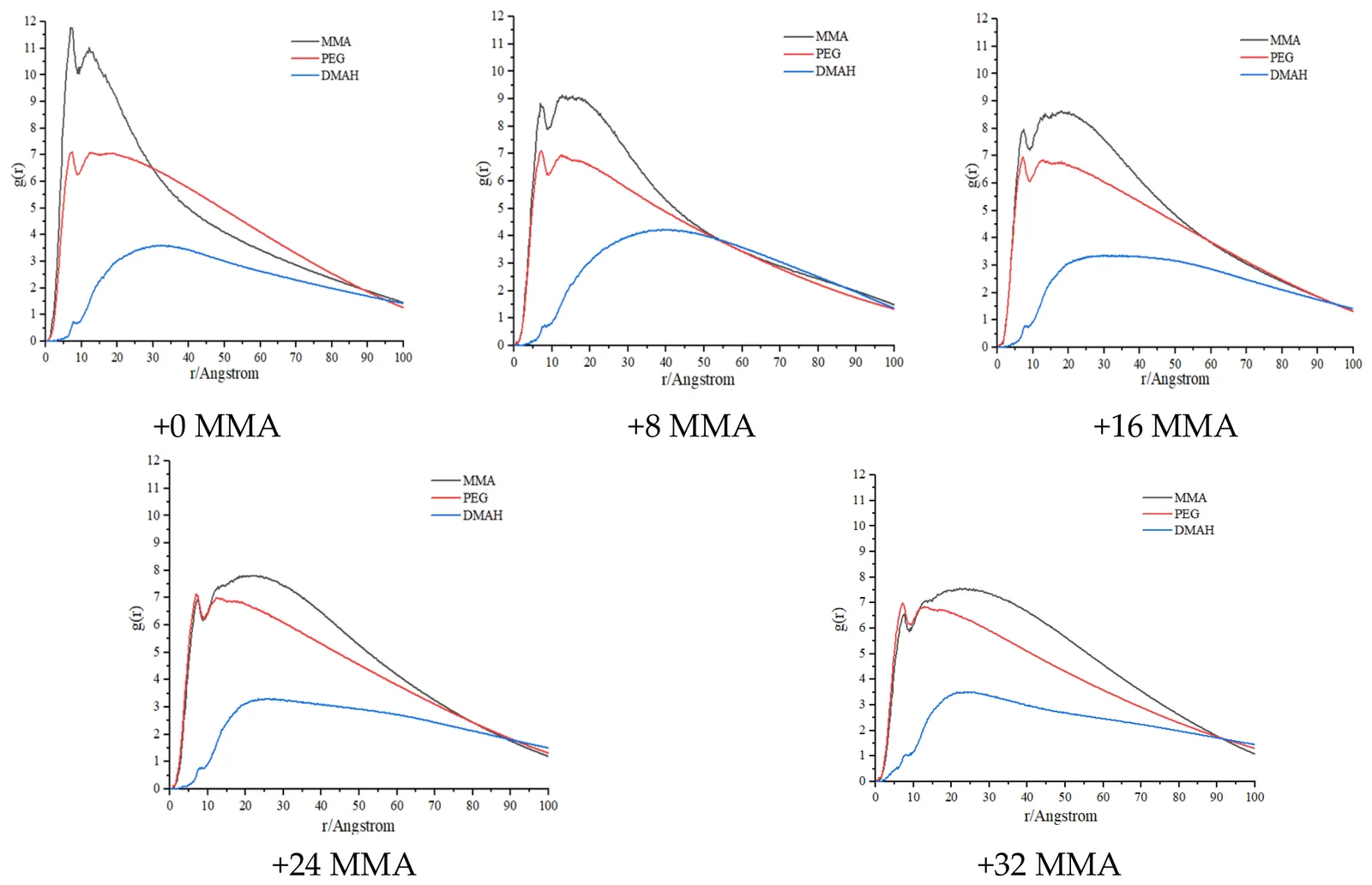
RDF curves of A/B mixed micelles with different hydrophobic block ratios.

To further investigate the effect of different hydrophobic block ratios on the micellar structure after protonation, RDF analysis was performed on five groups of A/B mixed micelles in Series I with varying MMA lengths (+0, +8, +16, +24, and +32 MMA), as shown in Figure 4.

The RDF curves reveal that as the MMA block ratio increased, the peak intensity of DOX-MMA RDF in the range of 10–20 Å exhibited a trend of initially decreasing and then leveling off, while the DOX-MMA curves gradually approached the DOX-PEG curves. This indicates a decrease in the spatial packing density between DOX and the MMA core, suggesting that DOX gradually moved away from the hydrophobic core region—implying drug release from the micellar interior toward the exterior. Notably, when the MMA content increased beyond +16, the changes in the RDF curves reached saturation, indicating that the micellar swelling reached a certain extent and the structure became stable, with further increases in hydrophobic block ratio producing only limited additional structural changes.

This phenomenon can be understood from the perspective of “structural resistance”: an appropriate increase in hydrophobic blocks enhances the hydrophobic interactions within the micellar core, facilitating more effective swelling and drug release after protonation. However, when the hydrophobic blocks are excessive, the core becomes overly dense, increasing the steric hindrance for drug molecules to traverse the core and thereby restricting further drug release [19]. This finding suggests that the effect of hydrophobic block ratio on drug release is not monotonically increasing but rather exhibits an optimal regime.

### 2.3. Effect of pH-Sensitive Block Ratio on Micellar Stability and Drug Loading Capacity

#### 2.3.1. Micellar Structural Stability

**Figure 5:**
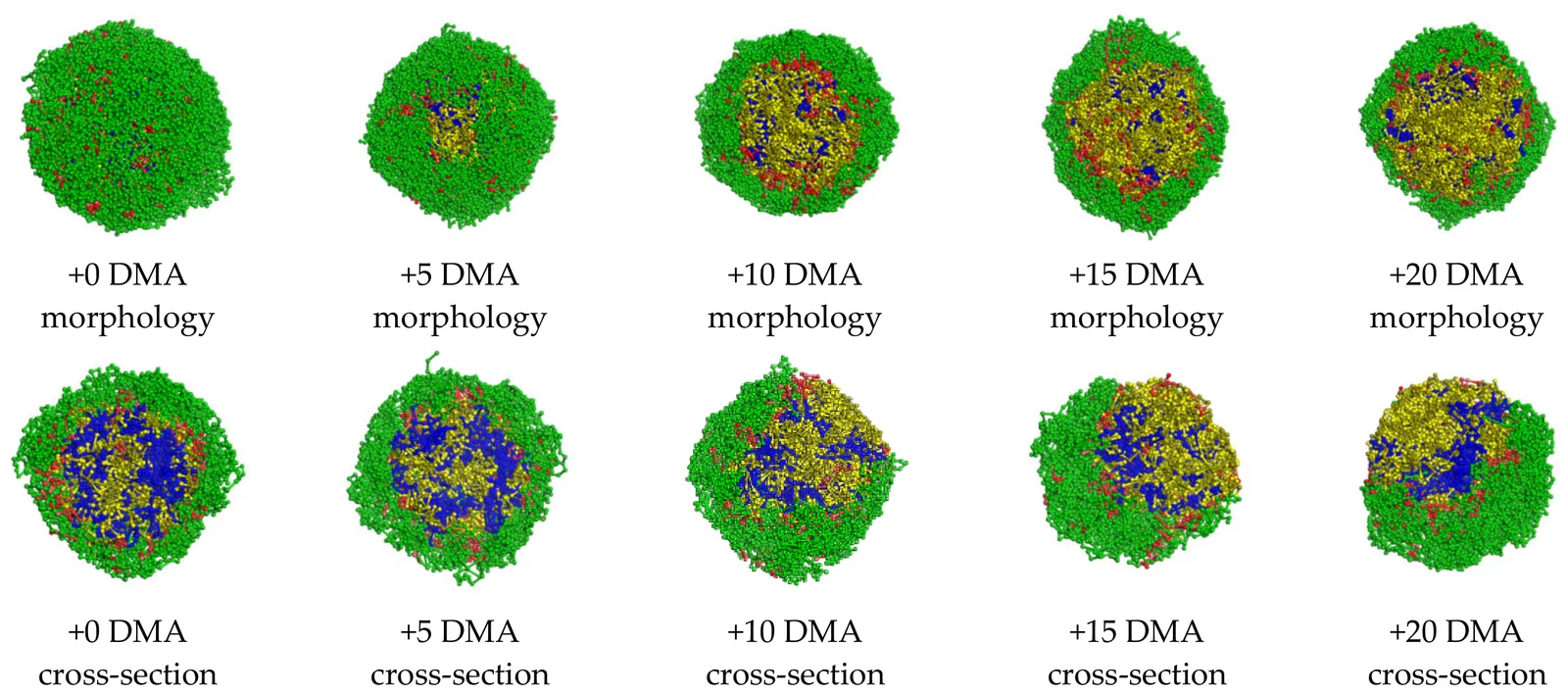
Morphology and cross-sectional views of A/B drug-loaded micelles with increasing pH-sensitive block ratios at pH 7.4.

**Figure 6:**
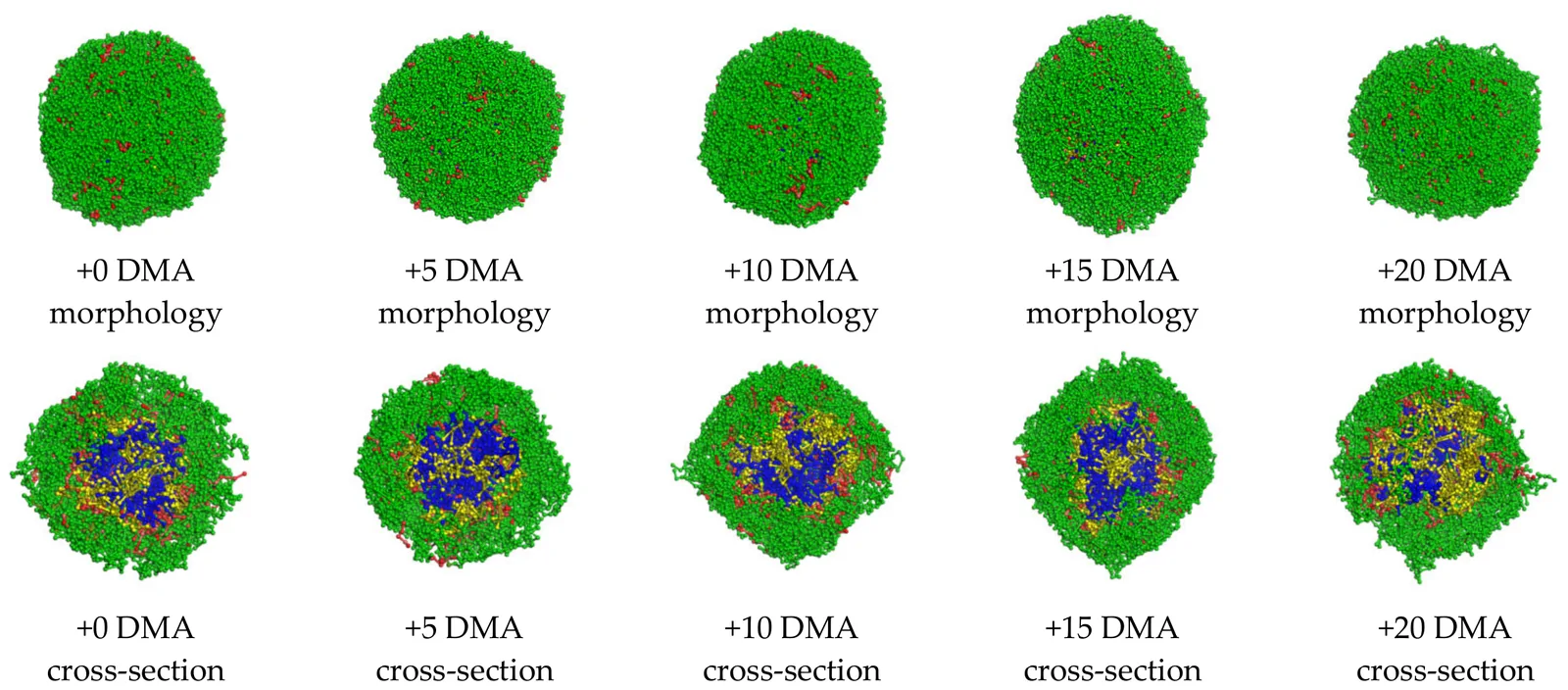
Morphology and cross-sectional views of A/C drug-loaded micelles with increasing pH-sensitive block ratios at pH 7.4.

The morphological analysis of A/B and A/C mixed micelles with five different DMA lengths in Series II (+0, +5, +10, +15, and +20 DMA) is shown in Figure 5 and Figure 6. As the DMA block ratio in polymers B and C increased, the structural stability of the A/B mixed micelles gradually declined. When the DMA content increased from +0 to above +15, some DMA blocks could no longer be effectively encapsulated by the PEG corona and became exposed on the micellar surface, compromising micellar integrity. In contrast, the A/C mixed micelles maintained a relatively intact core–shell structure even when the DMA content was increased to +20 [8].

**Figure 7:**
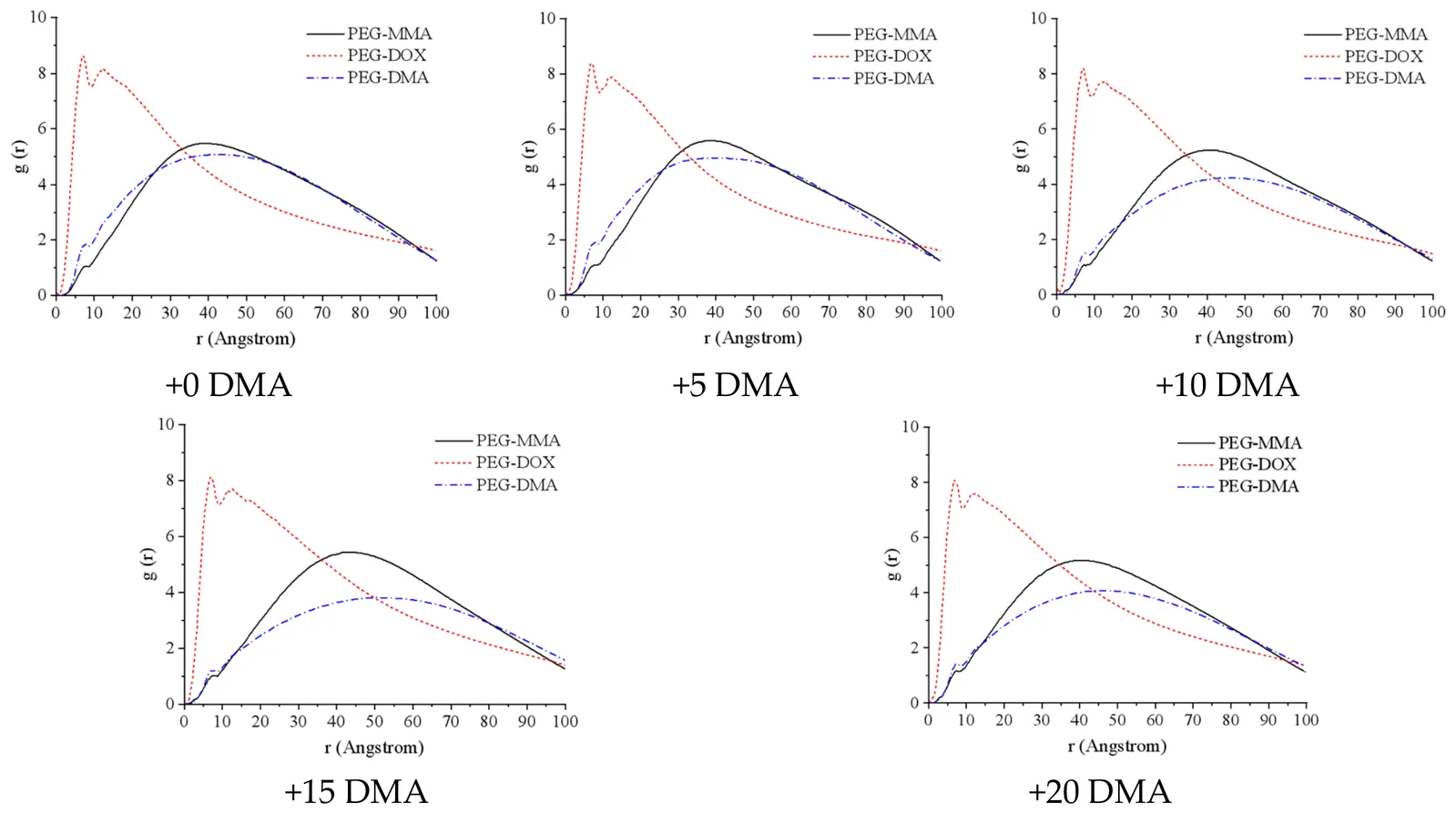
RDF curves of A/B drug-loaded micelles.

**Figure 8:**
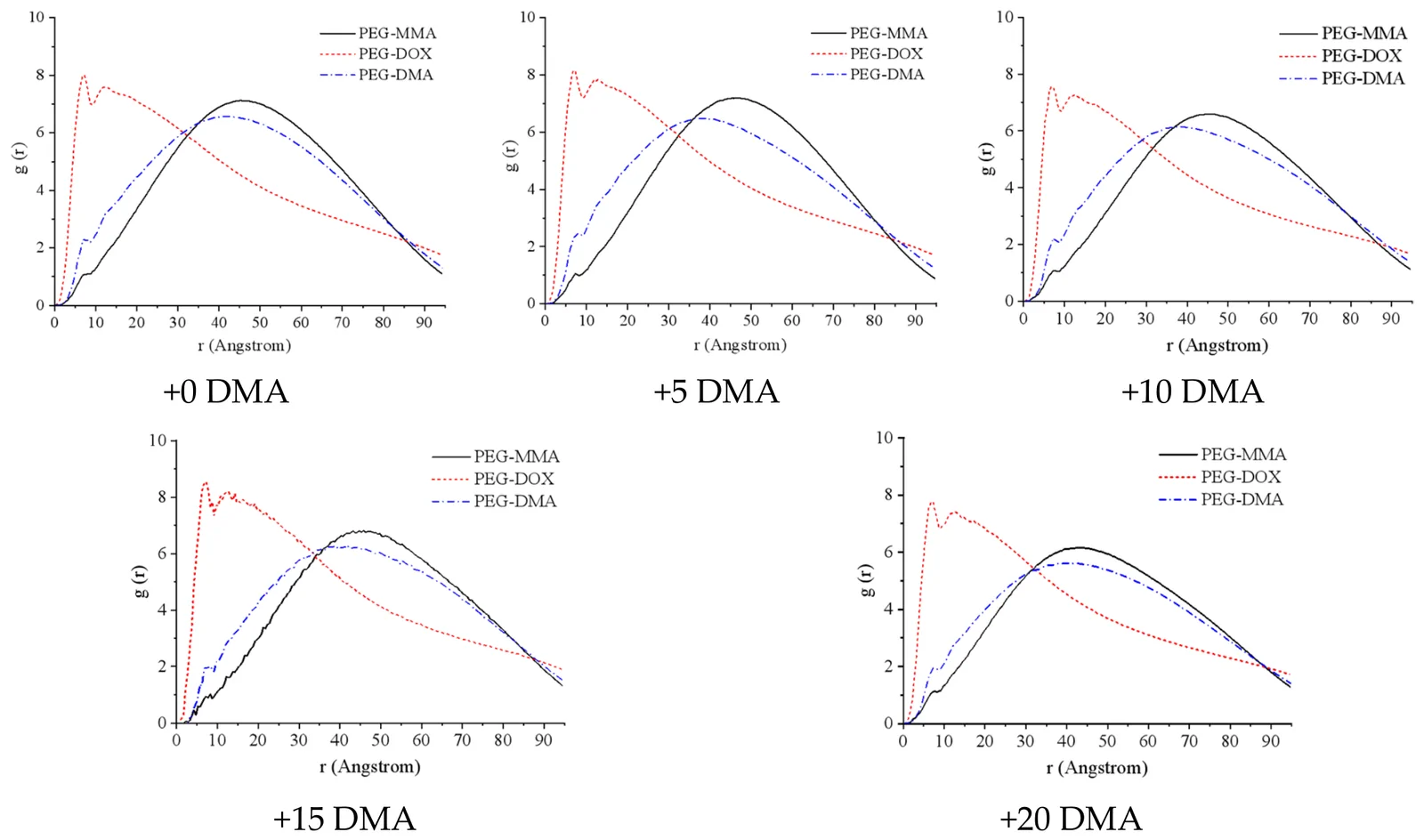
RDF curves of A/C drug-loaded micelles.

RDF analysis further confirmed this difference. As shown in Figure 7, in the A/B system, the peak intensity of the PEG-DMA RDF gradually decreased with increasing DMA ratio, and the PEG-DMA curves progressively deviated from the PEG-MMA curves, indicating that the DMA blocks gradually migrated outward from the protection of the PEG corona. In contrast, as shown in Figure 8, in the A/C system, the PEG-DMA and PEG-MMA RDF curves remained close to each other throughout, suggesting that the DMA blocks were effectively confined within the micellar shell [18].

The underlying reason for the above difference is that polymer B does not contain a hydrophilic PEG block. As the hydrophobic DMA blocks increase, the hydrophobic volume of the micellar core expands, while the accommodating capacity of the PEG corona becomes insufficient, leading to the exposure of DMA blocks [7]. In contrast, polymer C contains a substantial amount of PEG, which provides sufficient corona capacity to accommodate the increased DMA blocks, thereby endowing the A/C mixed micelles with superior structural stability. This result is consistent with the findings of Feng et al. [18] regarding the effect of hydrophilic block coverage on nanoparticle stability.

#### 2.3.2. Maximum Drug Loading Capacity

By gradually increasing the DOX proportion until the micelles could no longer fully encapsulate all drug molecules (with some drug molecules escaping into the aqueous phase), the maximum drug loading capacity of each system was determined [22], as presented in Table 3.

The results show that: (1) as the DMA block ratio increased, the maximum drug loading capacity of both A/B and A/C mixed micelles exhibited an upward trend. The A/B system increased from 9.09% at +0 DMA to 10.01% at +20 DMA (an average increase of 0.18% per 5 DMA units); the A/C system increased from 9.83% to 12.22% (an average increase of 0.49% per 5 DMA units) [20]. (2) The maximum drug loading capacity of the A/C mixed micelles was significantly higher than that of the A/B system at all DMA ratios.

These results can be explained from two aspects. On the one hand, the increase in DMA blocks thickens the intermediate layer of the micelles, providing a larger encapsulation space for hydrophobic drugs. On the other hand, the abundant PEG hydrophilic corona in the A/C system offers stronger structural support, preventing the thickening of the DMA layer from destabilizing the micellar structure and thus more effectively translating into enhanced drug loading capacity [5]. In contrast, the A/B system contains a higher proportion of hydrophobic MMA, and the thickening of the DMA layer simultaneously exacerbates the compactness of the core, partially offsetting the gain in encapsulation space. Therefore, the A/C mixed micelles, with their more stable structure, exhibit a distinct advantage in drug loading capacity.

### 2.4. Drug Release Kinetics: Synergistic Regulation of Block Ratios

#### 2.4.1. Effect of Hydrophobic Block Ratio on Release Rate

The mean square displacement (MSD) quantifies the average squared displacement of particles over time. A steeper MSD curve indicates more active diffusion, which in the context of micellar systems reflects the mobility of polymer blocks and the release of encapsulated drugs.

**Figure 9:**
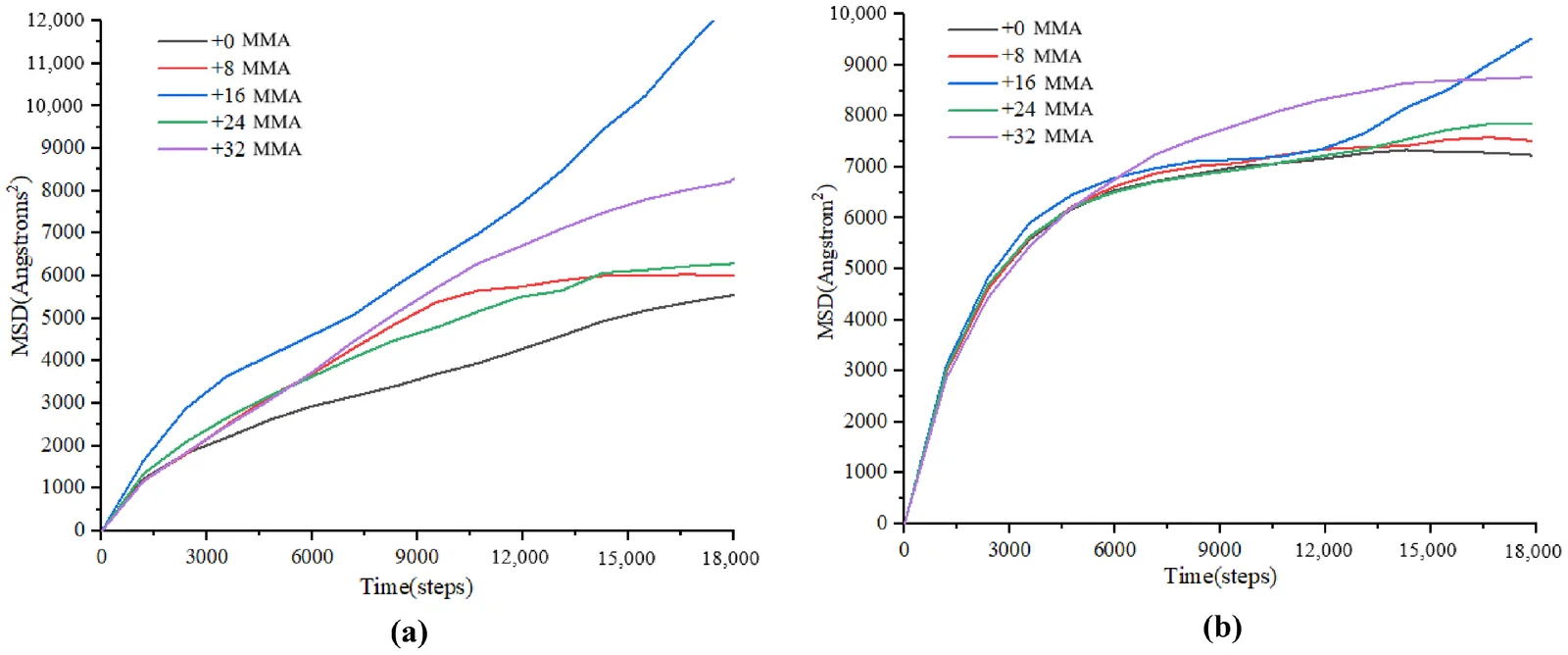
MSD curves of DMAH blocks in A/B and A/C mixed drug-loaded micellar systems in Series I. (**a**) A/B mixed drug-loaded micellar systems with varying MMA ratios. (**b**) A/C mixed drug-loaded micellar systems with varying MMA ratios.

By analyzing the MSD curves of DMAH and DOX under different MMA ratios in Series I [22] (see Figure 9), it was found that:

(1) The +16 MMA system exhibited the fastest increase in MSD of DMAH blocks, and the DOX release rate was also the fastest within the 0–6000 ns time window, indicating that the protonation response and drug release of the micelles were most active at this ratio [19].
(2) The +0 MMA system (initial state) showed the slowest MSD increase in DMAH blocks, and the DOX release rate was relatively slow and tended to level off over time, suggesting that micelles lacking sufficient hydrophobic blocks suffered from insufficient protonation driving force and limited swelling.
(3) When the MMA content was further increased from +16 MMA to +32 MMA, the MSD increase rates of both DMAH and DOX did not continue to rise but instead decreased, indicating that excessive hydrophobic blocks are detrimental to drug release [18].

This bell-shaped response pattern provides a clear quantitative basis for the optimal design of hydrophobic block ratios—in the present system, +16 MMA represents the optimal ratio. To rule out potential finite-size artifacts, we note that the simulation box (250 × 250 × 250 Å^3^) is substantially larger than the micellar diameter (~80–100 Å), ensuring minimal periodic interactions. The consistency of the +16 MMA optimum across both A/B and A/C systems, which differ in topological architecture and PEG corona thickness, further supports that this bell-shaped trend is an intrinsic feature of block ratio modulation rather than an artifact of finite-size effects or network topology constraints. The above results reveal a dual mechanism by which the hydrophobic block ratio regulates drug release: an appropriate amount of hydrophobic blocks enhances the hydrophobic interactions within the micellar core, leading to more intense structural reorganization at the core–shell interface upon protonation, thereby accelerating drug release. However, excessive hydrophobic blocks render the core overly dense, increasing the diffusion resistance for drug molecules and consequently suppressing release.

#### 2.4.2. Effect of pH-Sensitive Block Ratio on Release Rate

**Figure 10:**
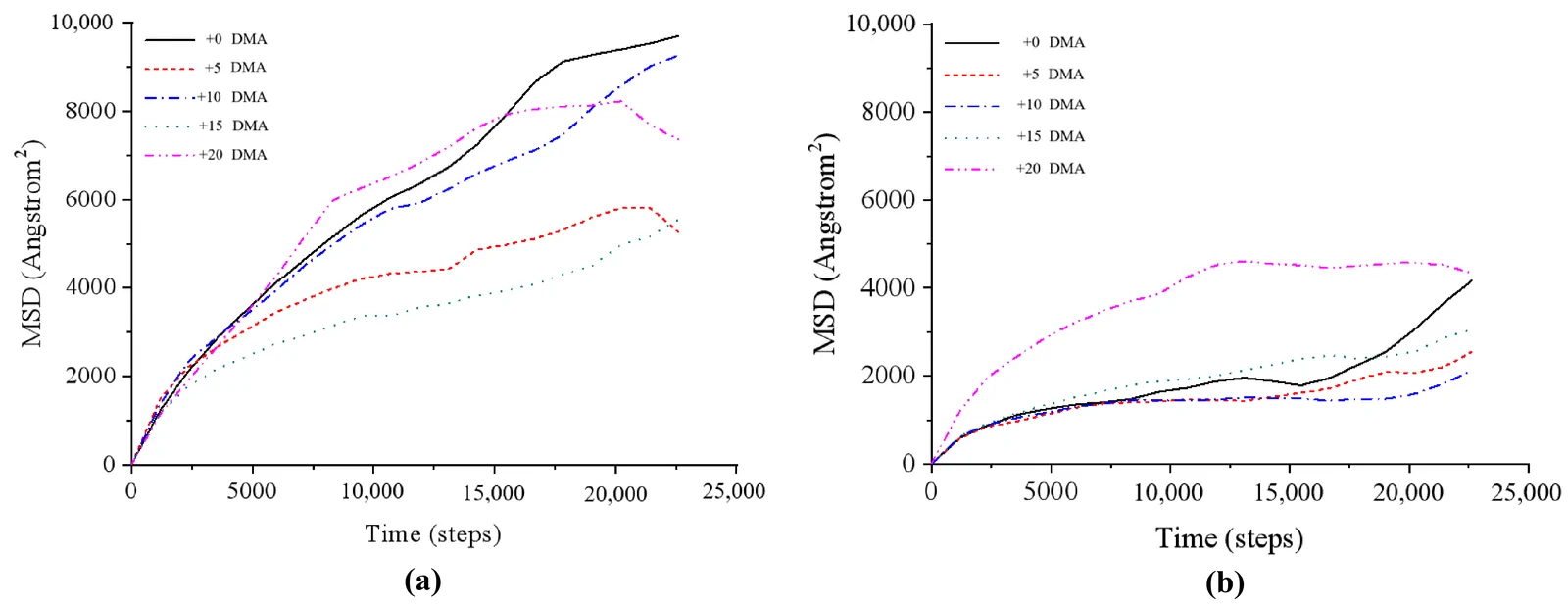
MSD curves of DMAH blocks in A/B and A/C mixed drug-loaded micellar systems in Series II. (**a**) A/B mixed drug-loaded micellar systems with varying DMA ratios. (**b**) A/C mixed drug-loaded micellar systems with varying DMA ratios.

MSD analysis was performed on the A/B and A/C systems with different DMA ratios in Series II (see Figure 10), and the results showed that:

(1) In the A/B system, the MSD of DMAH blocks exhibited a non-monotonic variation with increasing DMA ratio. The +0 DMA system showed relatively high DMAH mobility, which is attributed to its relatively higher PEG hydrophilic ratio, allowing water molecules to penetrate more easily into the micellar interior. The +20 DMA system also exhibited high DMAH mobility, possibly due to the stronger stretching driving force generated by the protonation of a higher proportion of DMA. In contrast, the systems with intermediate DMA ratios exhibited relatively lower mobility [8].
(2) In the A/C system, except for the +20 DMA system, in which the DMAH MSD curve increased markedly, the MSD curves of the +0 DMA to +15 DMA systems all exhibited relatively low mobility. This further confirms the buffering effect of the hydrophilic PEG corona on micellar swelling: in the A/C system, the thick PEG corona retards water penetration, thereby slowing down the protonation-driven micellar swelling process [7].

Comparison of the DOX MSD curves revealed that although increasing the DMA ratio significantly affected the micellar swelling rate, its influence on the total amount of drug release was relatively limited. The DOX MSD curves of all systems tended to converge to similar levels over the long term (>10,000 ns). This indicates that the primary role of the pH-sensitive block is to act as a “switch” triggering micellar swelling, while the ultimate efficiency of drug release depends more on the overall structural characteristics of the micelles (such as hydrophilic corona thickness and hydrophobic core compactness) [16].

#### 2.4.3. “Driving Force–Resistance” Competition Model

Based on the above results, this study proposes a “driving force–resistance” competition model for drug release from mixed micelles [21]:

(1) The protonation driving force is provided by the DMA blocks: the hydrophilic transition of DMAH upon protonation drives micellar swelling and structural dissociation, serving as the fundamental driving force for drug release. Increasing the DMA ratio can enhance this driving force, but is constrained by the structural accommodating capacity of the micelles [10].
(2) The structural resistance is jointly determined by the compactness of the hydrophobic core and the thickness of the hydrophilic corona. An appropriate amount of MMA enhances the hydrophobic interactions within the core, facilitating structural reorganization during swelling; however, excessive MMA increases the core density, forming a diffusion barrier [19]. Although a thick PEG corona can enhance micellar stability, it also retards water penetration and the micellar swelling rate [8].

The drug release rate depends on the competitive balance between the driving force and the resistance. In the A/B system, the higher proportion of hydrophobic MMA provides a stronger protonation driving force, but excessive MMA also introduces greater structural resistance, resulting in an optimal MMA ratio (+16 MMA). In the A/C system, the thicker hydrophilic PEG corona leads to greater structural resistance, so that even with increasing DMA ratio, the enhancement in release rate remains relatively limited; nevertheless, this also endows the A/C system with superior structural stability and higher drug loading capacity [7].

It is worth noting that the conformational flexibility of the PEG corona may differ between linear and star-like copolymer topologies. In linear architectures, PEG chains exhibit relatively free mobility, whereas star-like architectures may impose topological constraints that limit chain rearrangement and affect the buffering capacity of the corona. The present model framework remains applicable across these architectures, but the specific conformational characteristics of the corona should be considered when applying the model to different polymer topologies.

This model provides a clear theoretical framework for the rational design of pH-responsive mixed micelles: if the goal is to achieve rapid and efficient drug release at tumor sites, the A/B-type system with an appropriate hydrophobic block ratio should be prioritized; if the goal is to achieve high drug loading capacity and long-term circulation stability, the A/C-type system is more advantageous.

#### 2.4.4. Comparison and Validation with Experimental Studies

To verify the reliability of the above simulation results, this section systematically compares the simulation findings with experimental studies in the same field.

(1) Effect of hydrophobic block ratio on release rate: Comparison with the experimental results of Huang et al.

Huang et al. [20] experimentally synthesized mixed micelle systems composed of linear mPEG-b-PCL and star-shaped S(PCL-b-PDEAEMA), and systematically investigated the effects of different polymer composition ratios on the particle size and drug release behavior of the mixed micelles. Their experiments revealed that as the proportion of mPEG-b-PCL increased (i.e., with increasing hydrophilic component content), the micellar size gradually decreased, and significant differences in in vitro drug release rates were observed. These experimental results are consistent with the trends revealed by our simulations: the higher PEG ratio in the A/C system forms a thicker hydrophilic corona, which retards water penetration and micellar swelling, resulting in a relatively slower drug release rate; whereas the A/B system, with a higher proportion of hydrophobic MMA, responds more rapidly to pH changes and exhibits a faster release rate. Huang et al. also pointed out that the drug release behavior of mixed micelles is the result of the coupling among “polymer composition–micellar structure–release performance,” which is conceptually highly consistent with the “driving force–resistance” competition model proposed in this study.

(2) Effect of pH-sensitive block ratio on drug loading capacity: Comparison with the experimental and simulation studies of Yang et al.

Yang et al. [14] employed both experimental synthesis and DPD simulation methods to investigate the drug loading and release performance of PDEAEMA-PPEGMA and PCL-PPEGMA mixed micelles. Their experimental results showed that as the proportion of PDEAEMA (i.e., DMA) increased, both the drug loading capacity (LC) and encapsulation efficiency (EE) of the micelles exhibited upward trends, and the DPD-simulated drug distribution profiles confirmed the increased enrichment of DOX within the micellar interior. The magnitude of drug loading enhancement in that study is in full agreement with the trend observed in our simulation results (from 9.83% to 12.22% for the A/C system). Yang et al. attributed this phenomenon to the thickening of the pH-sensitive block, which provides a larger encapsulation space for hydrophobic drugs, corroborating our interpretation from the perspective of “DMA layer thickening.”

Furthermore, both the in vitro release experiments and DPD simulations of Yang et al. [14] demonstrated that the mixed micelles exhibited good stability under neutral conditions (with minimal drug leakage), while showing accelerated release behavior under weakly acidic conditions (accompanied by a small initial burst release). This “pH-triggered release” behavior pattern is fully consistent with the mechanism observed in our study—protonation-induced micellar swelling and structural loosening leading to enhanced drug release—confirming that DPD simulations can effectively capture the key physical features of pH-responsive micellar release behavior.

(3) Protonation-induced micellar structural transition: Comparison with the experimental and simulation studies of Wang et al. (2015).

Wang et al. (2015) [21] employed DPD simulations combined with experimental methods to investigate the microstructural changes of histidine-modified pH-sensitive polypeptide micelles (DHA-His_x-Lys_10) under different pH conditions. Their simulation results showed that at pH > 6.0, the micelles exhibited a dense and compact structure with drugs well-encapsulated within the micellar core; when the pH decreased to 5.0, the histidine-containing micelles underwent a structural transition from a dense state to a swollen state, with micellar size increasing from 10.3 to 14.5 DPD units. This “dense → swollen” structural transition pattern is completely consistent with our observations of protonated micelles evolving from well-defined layered structures to internally cracked and loosened structures. The in vitro release experiments in that study further confirmed that decreasing pH from 7.4 to 5.0 significantly accelerated DOX release, validating that the simulated structural transition indeed promoted drug release.

Wang et al. (2015) [21] specifically noted in their paper that “the integration of simulation and experiment may be a valuable approach for optimizing and designing biomedical materials with desired properties.” The present study, precisely within this methodological framework, has revealed the molecular mechanisms of block ratio-modulated micellar performance through systematic DPD simulations, forming a good complement to the conclusions of the aforementioned combined experimental–simulation studies.

(4) Buffering effect of the hydrophilic corona: Comparison with experimental studies on mixed micelles

Zhao et al. [23] recently reported a study on folate-modified pH-responsive copolymer mixed micelles for anticancer drug delivery. Their experiments revealed that the PEG-containing mixed copolymer micelles (P1/P2) exhibited superior comprehensive drug loading performance and drug controlled-release performance compared to single copolymer micelles, with a critical micelle concentration (CMC) as low as 2.51 mg/L, indicating that the PEG hydrophilic corona significantly enhances micellar stability. These findings are highly consistent with our conclusion that “the hydrophilic PEG blocks in the A/C system provide structural stability and retard drug release,” further validating the key role of hydrophilic corona thickness in regulating micellar stability and release kinetics.

(5) Discussion on model applicability

The above experimental comparisons demonstrate that the “driving force–resistance” competition model proposed in this study is not only applicable to PDEAEMA-based mixed micellar systems but may also be extended to other pH-responsive micellar systems. Specifically:

(1) For systems containing weakly basic polyelectrolytes (e.g., PAE-, PDEA-, and histidine-containing micelles): the protonation-induced hydrophobic-to-hydrophilic transition is the core mechanism triggering release, and our model can be directly applied. The “dense → swollen” transition observed by Wang et al. (2015) [21] in histidine-modified micelles, as well as the phenomenon of PAE-based micelles undergoing structural dissociation due to enhanced electrostatic repulsion under acidic conditions [8], are both consistent with the “protonation driving force” mechanism proposed in this study.
(2) For systems containing weakly acidic polyelectrolytes (e.g., PAA-containing micelles): the carboxyl groups of PAA become protonated and more hydrophobic under acidic conditions, with the response direction opposite to that of basic systems. Nevertheless, the “driving force–resistance” analytical framework remains applicable—the driving force originates from chain conformational changes and hydrophobic aggregation induced by carboxyl protonation, while the resistance similarly arises from core densification and corona barrier effects [8].
(3) Applicability conditions and limitations of the model: The validity of this model depends on the following conditions: ① the micelles possess a well-defined core–shell–intermediate layer three-layer structure; ② the pKa of the pH-sensitive block matches the target responsive pH window; and ③ the compactness of the hydrophobic core is moderate, allowing protonation-driven structural reorganization to occur effectively. When the hydrophobic block ratio is excessively high, leading to a glassy or crystalline state of the core, structural resistance will dominate the release behavior, and the bell-shaped response pattern may no longer hold. Furthermore, the present model does not account for the fine-tuning of release kinetics by specific drug–polymer interactions (e.g., hydrogen bonding, π–π stacking), which represents a direction for further investigation in future studies.

The above experimental comparisons demonstrate that the DPD simulation results are in good agreement with experimental studies in the same field, validating the reliability of our simulation methodology and the rationality of the “driving force–resistance” model. Meanwhile, the discussion on the applicability of this model to different pH-responsive micellar systems also provides a testable theoretical framework for future research. The cross-sectional morphologies predicted by the simulations (Figure 5, Figure 7 and Figure 8) could be validated experimentally using cryogenic transmission electron microscopy (cryo-TEM), which has been successfully employed to image the internal structure of polymeric micelles with nanometer resolution. However, the transient nature of the protonation process and the challenges of sample preparation for cryo-TEM observation of dynamic structural changes pose significant experimental difficulties, which motivated the use of DPD simulations in the present study.

## 3. Conclusions

In this study, dissipative particle dynamics simulations were employed to systematically investigate the effects of hydrophobic block (MMA) and pH-sensitive block (DMA) ratios on the protonation-responsive behavior, structural stability, drug loading capacity, and drug release performance of PDEAEMA-containing mixed polymeric drug-loaded micelles. The main conclusions are as follows:

(1) The hydrophobic block ratio regulates the protonation response and release kinetics. Increasing the hydrophobic block ratio accelerates the protonation-triggered micellar swelling and the outward extension of DMAH blocks. However, the drug release rate exhibits a non-monotonic dependence on the MMA ratio, with an optimal ratio (+16 MMA) identified in this system. Excessive hydrophobic blocks increase steric hindrance due to core densification, thereby suppressing drug release [18,19]. This bell-shaped response pattern provides a quantitative basis for the optimal design of hydrophobic block ratios.
(2) The pH-sensitive block ratio modulates drug loading capacity and structural stability. Increasing the DMA block ratio significantly enhances the maximum drug loading capacity of the mixed micelles, from 9.83% to 12.22% for the A/C system and from 9.09% to 10.01% for the A/B system [20]. However, increasing the DMA ratio compromises the structural stability of the A/B system (due to the lack of sufficient PEG corona to accommodate the enlarged hydrophobic core), while exerting only a minor effect on the stability of the PEG-rich A/C system [8]. The hydrophilic PEG block plays a critical role in maintaining micellar structural integrity.
(3) The drug release from mixed micelles is governed by the competing mechanism of “protonation driving force versus structural resistance” [21]. DMA provides the protonation driving force, while MMA and PEG jointly determine the structural resistance. The A/B system exhibits a strong driving force but relatively weak stability, making it suitable for rapid release scenarios; the A/C system offers high stability and drug loading capacity but slower release, making it more appropriate for long-circulation scenarios [7]. Each system has its own advantages, and the choice should be made according to specific therapeutic requirements.
(4) DPD simulation provides an effective mesoscopic tool for the rational design of pH-responsive mixed micelles [11,12]. The “block ratio–micellar structure-drug release performance” relationships established in this study can provide theoretical guidance for the polymer composition design of novel pH-responsive mixed micelles in experimental research, helping to reduce trial-and-error costs and accelerate the development of high-performance drug delivery vehicles.

It should be acknowledged that the coarse-grained representation of DOX as three bead types (D1, D2, D3) does not explicitly capture specific secondary interactions such as hydrogen bonding or π–π stacking between DOX and the polymer blocks. While this simplification is appropriate for capturing the mesoscale self-assembly and structural transitions that are the primary focus of this study, future work incorporating higher-resolution models could further elucidate the role of specific drug–polymer interactions in modulating loading and release behavior.

Furthermore, the self-assembly principles and pH-responsive structural transition mechanisms elucidated in this micellar system are expected to provide valuable references for the design of gel-based drug delivery systems. Polymeric micelles and gels are closely interconnected as hierarchical soft material systems, where micellar self-assembly represents a fundamental building principle—micellar aggregates can serve as crosslinking nodes or structural units in supramolecular hydrogels. The structure–performance relationships and block ratio modulation strategies established in this study therefore offer molecular-level insights that can be extended to gel-related soft materials engineering, where similar block copolymers are frequently employed as building blocks for constructing hydrogels with tunable release profiles [24,25].

While the present study is focused on mesoscale simulations, the good agreement between our results and experimental findings reported in the literature [14,20,22,23] supports the reliability of our simulation methodology. Future experimental validation, for instance using cryogenic transmission electron microscopy (cryo-TEM) to directly image the internal structure of micelles, would further strengthen the findings and is a direction we are actively pursuing in our ongoing research.

## 4. Materials and Methods

### 4.1. Research Systems

Three polymers were employed in this study: a triblock copolymer mPEG-b-PDEAEMA-b-PMMA (polymer A), a diblock copolymer PDEAEMA-b-PMMA (polymer B), and a diblock copolymer PPEGMA-b-PDEAEMA (polymer C). The chemical structures of the three polymers are illustrated in Figure 1. According to preliminary experimental results, a mass ratio of 8:2 for polymer A to polymer B (or C) yielded mixed micelles with stable core–shell structures under the simulation conditions; therefore, this ratio was fixed throughout the study to isolate the effects of other block length variations on micellar performance. Doxorubicin (DOX) was selected as the model anticancer drug. All three polymers contain a PDEAEMA (DMA) block to confer pH responsiveness. Polymer A additionally contains both a PEG block and an MMA block; polymer B contains an MMA block but no PEG block; and polymer C contains a PEG block but no MMA block.

**Figure 11:**
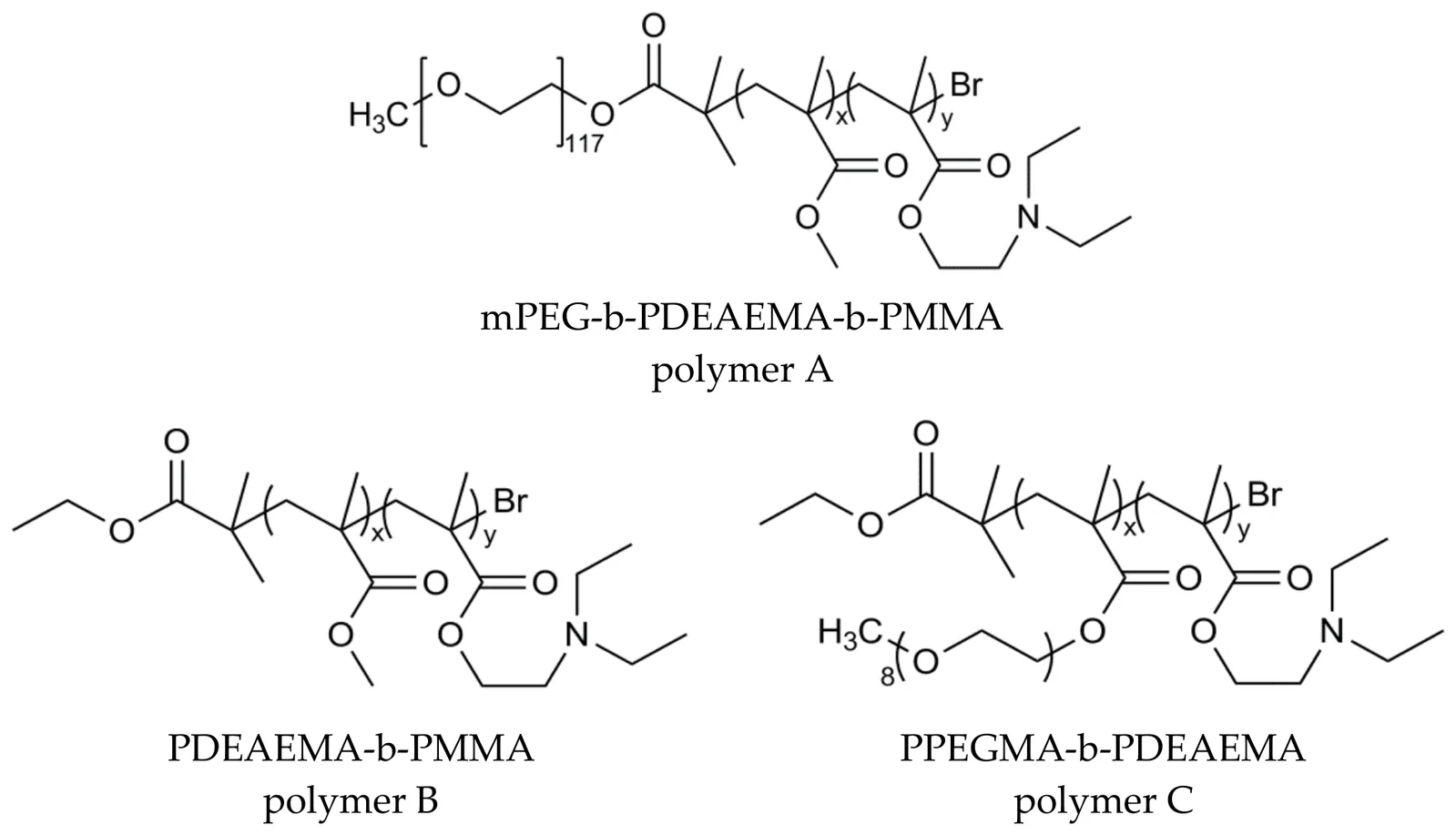
Schematic representation of the chemical structures of polymers A, B, and C.

In Figure 11, the initial values of x (MMA units) and y (DMA units) are x = 7 and y = 15, respectively. The notation ‘+n MMA’ or ‘+n DMA’ indicates the number of additional units added to the respective block. For instance, ‘+16 MMA’ corresponds to an MMA block length of 7 + 16 = 23 units.

To systematically investigate the influence of block ratios, two series of simulations were designed: (1) Series I—the compositions of polymers B and C were kept constant, while the hydrophobic block length in polymer A was increased by increments of 8 MMA units (+0, +8, +16, +24, and +32 MMA). Both A/B and A/C mixed micellar systems were examined in this series. (2) Series II—the composition of polymer A was kept constant, while the pH-sensitive block length in polymers B and C was increased by increments of 5 DMA units (+0, +5, +10, +15, and +20 DMA). Again, both A/B and A/C mixed micellar systems were investigated in this series.

### 4.2. Dissipative Particle Dynamics Method

In the DPD method, each particle represents a coarse-grained unit comprising multiple atoms or molecules [11]. The motion of the particles is governed by Newton’s equations of motion:

(1) $$\frac{dr_{i}}{dt}=v_{i},\;\;m_{i}\frac{dv_{i}}{dt}=f_{i}$$

where r_i_, v_i_, m_i_, and f_i_ are the position vector, velocity, mass, and resultant force of particle i, respectively. The total force acting on each particle consists of three components: the conservative force F_ij_^C^, the dissipative force F_ij_^D^, and the random force F_ij_^R^ [26]:

(2) $$f_{i}=\sum_{i\neq j}(F_{ij}^{C}+F_{ij}^{D}+F_{ij}^{R})$$

The three forces are explicitly expressed as follows [27,28]:

(3) $$F_{ij}^{C}=\left\{\begin{array}{c} a_{ij}\left(1-r_{ij}\right){\hat{r}}_{ij}\;\;\;\;(r_{ij}<1)\\ \;\;\;\;\;\;0\;\;\;\;\;\;\;\;\;\;(r_{ij}\geq 1) \end{array}\right.$$

(4) $$F_{ij}^{D}=\left\{\begin{array}{c} -\gamma \omega^{D}\left(r_{ij}\right)\left(\hat{r}\cdot v_{ij}\right){\hat{r}}_{ij}\;\;\;\;(r_{ij}<1)\\ \;\;\;\;\;\;\;\;\;0\;\;\;\;\;\;\;\;\;\;\;\;(r_{ij}>1) \end{array}\right.$$

(5) $$F_{ij}^{R}=\left\{\begin{array}{c} \sigma \omega^{R}\left(r_{ij}\right)\theta_{ij}{\hat{r}}_{ij}\;\;\;\;\;(r_{ij}<1)\\ \;\;\;\;\;\;0\;\;\;\;\;\;\;\;\;\;(r_{ij}>1) \end{array}\right.$$

where a_ij_ is the maximum repulsion parameter between particles i and j, r_ij_ is the interparticle distance, γ is the dissipation strength, σ is the noise amplitude, ω^D^ and ω^R^ are the weight functions, and θ_ij_ is a random variable satisfying a Gaussian distribution. The weight functions satisfy the following relations [29]:

(6) $$\sigma =\sqrt{2\gamma k_{B}T}$$

(7) $$\omega^{R}\left(r_{ij}\right)=1-r_{ij}/R_{c}$$

(8) $$\omega^{D}\left(r_{ij}\right)={\left[\omega^{R}\left(r_{ij}\right)\right]}^{2}={\left[1-r_{ij}/R_{c}\right]}^{2}$$

The weight functions ω^D^ and ω^R^ are given by Equations (7) and (8), respectively, and satisfy the fluctuation–dissipation theorem as expressed in Equation (6), where R_c_ is the cutoff radius. Adjacent particles along the same polymer chain are connected by a harmonic spring force [29]:

(9) $$F_{i}^{S}=\sum_{j}\;Cr_{ij}$$

where C is the spring constant. All DPD simulations were performed using the Mesocite module (BIOVIA, San Diego, CA, USA) as implemented in the Materials Studio software package.

### 4.3. Coarse-Grained Model and Simulation Parameters

**Figure 12:**
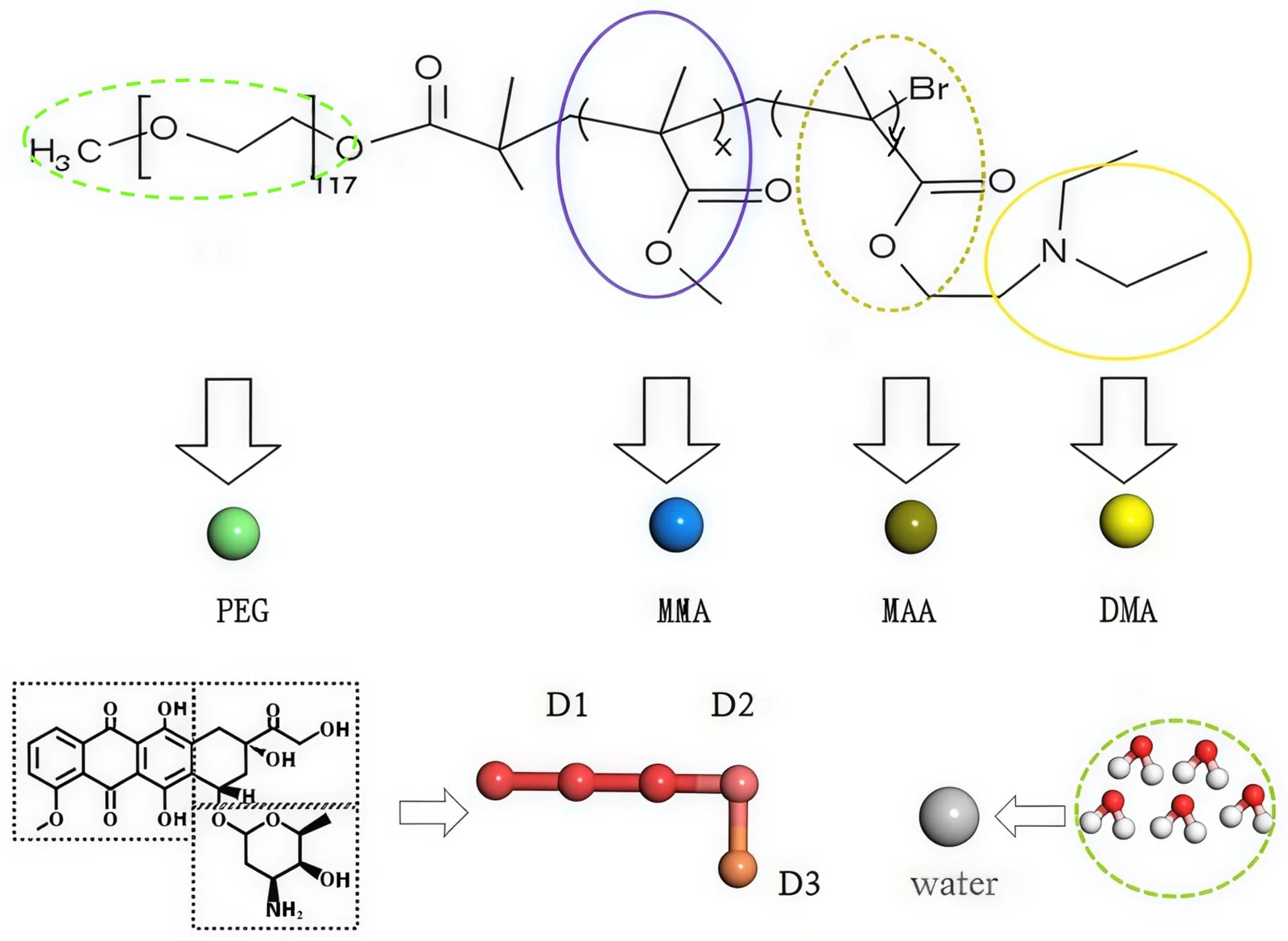
Molecular structures and coarse-grained models of each component in the simulated systems.

According to the physicochemical properties (hydrophilicity, hydrophobicity, etc.) of each component, the polymers were partitioned into distinct beads (as shown in Figure 12). The PEG block is represented by green beads, the MMA block by blue beads, the DMA block (which becomes DMAH after protonation) by yellow beads, and the MAA component linking the MMA and DMA blocks by brown beads. In the coarse-grained model, the ester linkage connecting the DMA and MMA blocks is represented as a separate bead type (denoted as MAA) to preserve the connectivity and physicochemical properties of the polymer backbone. This bead type does not represent a free methacrylic acid monomer but rather the connecting unit within the polymer chain. The DOX molecule is divided into three types of beads, namely D1, D2, and D3 (colored red, pink, and deep pink, respectively), while water molecules are represented by gray beads [30].

The interaction parameter a_ij_ between beads was calculated from the Flory–Huggins parameter χ_ij_ [31,32]:

(10) $$a_{ij}=\chi_{ii}+3.27\chi_{ij}$$

where a_ii_ = 25 (corresponding to k_B_T = 1), and χ_ij_ was calculated from the solubility parameters δ of each component [33]:

(11) $$\chi_{ij}=\frac{(\delta_{i}-\delta_{j}{)}^{2}V}{RT}$$

The resulting repulsion parameters reflect the strong hydrophobicity of the unprotonated DMA block; while the DMA-water a_ij_ value (115.27) is higher than typical for hydrophilic polymers, it is consistent with the solubility parameter differences for this highly hydrophobic system and has been validated against literature values for similar polymer-water systems. The corresponding χ parameter (χ ≈ 27.6) reflects the strong hydrophobicity of the unprotonated DMA block. Control simulations with a reduced repulsion parameter (a_ij_ = 80) resulted in diffuse, unstable micellar structures that failed to maintain a stable core–shell morphology, confirming that the present value (a_ij_ = 115.27) does not introduce artificial phase separation and best reproduces the expected micellar behavior consistent with experimental observations.

The simulation box dimensions were set to 250 × 250 × 250 Å^3^, and the volume ratios of polymer, DOX, and water were set to 10:4:86 (for Series I) or 10:1:89 (for Series II). The time step was set to 0.05 ns, and the total number of simulation steps ranged from 150,000 to 200,000 steps. The degree of protonation was calculated using the following equation [21]:

(12) $$\alpha_{H}^{+}=\frac{1}{1+10^{pK_{a}-pH}}$$

The pKa of the DMA block in solution is approximately 7.0–7.4, but in the polymer brush environment, the effective degree of protonation is lower than in free solution due to counterion concentration effects. At pH 5.0, the estimated protonation degree is approximately 97%, consistent with literature reports for similar systems.

### 4.4. Analysis Methods

The following analytical methods were employed to characterize the micellar structure and drug release behavior in this study:

Radius of Gyration (R_g_) was used to characterize the extent of polymer chain extension in space [34]:

(13) $${R_{g}}^{2}\;=\;(\frac{1}{N})\;\sum_{i=1}N\;{\left|r_{i}\;-\;r_{\mathrm{cm}}\right|}^{2}$$

where r_cm_ is the position of the center of mass.

The radial distribution function (RDF) was used to analyze the spatial distribution relationship between different components [34]:

(14) $$g_{ij}\left(r\right)=\frac{\langle ∆N_{ij}\left(r\to r+∆r\right)\rangle V}{4\pi r^{2}∆rN_{i}N_{j}}$$

where $\langle ∆N_{\mathrm{ij}}\left(r\to r+∆r\right)\rangle$ is the average number of j-beads within a spherical shell from distance r to r + Δr around bead i, N_j_ and N_i_ are the numbers of j-beads and i-beads, respectively, and V is the total volume of the system.

The mean square displacement (MSD) was used to characterize the diffusion behavior of particles [34]:

(15) $$MSD=\frac{1}{N}\sum_{i=1}^{N}{\left|r_{i}\left(t\right)-r_{i}\left(0\right)\right|}^{2}$$

where r_i_ is the position vector of bead i, and N is the total number of beads in the polymer. The DPD simulation results were mapped to physical units using a length scale of Rc ≈ 6.46 Å per bead (corresponding to three water molecules per DPD bead) and a time scale of 0.05 ns per DPD step, consistent with previous DPD studies of similar polymeric systems [3,4,34]. Therefore, the MSD values in Figure 11 and Figure 12 are reported in Å^2^. All statistical analyses and plotting were performed using OriginLab (OriginLab Corporation, Northampton, MA, USA). We confirm that the same conversion scales (Rc ≈ 6.46 Å per bead and Δt = 0.05 ns per DPD step) were applied uniformly to all MSD curves in both Series I and Series II, ensuring dynamic comparability across all systems.

## Figures and Tables

**Table 1:** Comparison of interaction parameters of the pH-sensitive block before and after protonation.

	MMA	MAA	PEG	D1	D2	D3	Water
DMA	25.65	37.87	36.56	28.36	26.17	26.53	115.27
DMAH	232.19	143.10	137.94	200.78	252.76	226.82	31.19

**Table 2:** Radius of gyration of the pH-sensitive block before and after protonation.

Micelle	pH	Radius	pH	Radius
A/B	7.4	19.72	5.0	28.25
A/C	7.4	18.3	5.0	23.3

**Table 3:** Maximum drug loading capacity of the ten types of A/B and A/C drug-loaded micelles.

**A/B Mixed micelles**	**+0 DMA**	**+5 DMA**	**+10 DMA**	**+15 DMA**	**+20 DMA**
Drug loading capacity	9.09%	9.73%	9.82%	9.92%	10.01%
**A/C Mixed micelles**	**+0 DMA**	**+5 DMA**	**+10 DMA**	**+15 DMA**	**+20 DMA**
Drug loading capacity	9.83%	10.62%	11.79%	11.88%	12.22%

## Data Availability

The original contributions presented in the study are included in the article, and further inquiries can be directed to the corresponding authors.
